# Fluorescence Spectra of Prototropic Forms of Fluorescein and Some Derivatives and Their Potential Use for Calibration-Free pH Sensing

**DOI:** 10.3390/s24051705

**Published:** 2024-03-06

**Authors:** Bernard Gauthier-Manuel, Chafia Benmouhoub, Bruno Wacogne

**Affiliations:** 1 CNRS, Institut FEMTO-ST, Université de Franche-Comté, 25000 Besançon, France; bernardgauthier55@gmail.com (B.G.-M.); benmouhoubc@gmail.com (C.B.); 2 SATT Grand Est, SAYENS, 25000 Besançon, France; 3 INSERM CIC 1431, Besançon University Hospital, 25030 Besançon, France

**Keywords:** fluorescence pH sensing, fluorescein, FITC, FAM, spectrum modeling

## Abstract

Fluorescence pH sensing has proven to be efficient but with the drawback that molecules photobleach, requiring frequent calibrations. Double-emission peak molecules allow ratiometric measurements and theoretically avoid calibration. However, they are often expensive and fragile and usually have very low quantum yields. Single emission peaks such as fluorescein and derivatives are inexpensive and have very high quantum yields. Because they are single emission peaks, the pH is assumed to be derived from the ratio of emitted intensities at measured pH and at high pH values, i.e., they require frequent calibration. However, the shape of their single emitted peak evolves slightly with pH. In this paper, we first demonstrate a simple method to calculate the emission spectrum shape of each prototropic form of fluorescein (and derivatives) as well as the values of the pKa_s_. A complete model of the evolution of the emission spectrum shape with pH is then constructed. Second, we evaluate the potential of these molecules for pH sensing by fitting the experimental spectra with the complete emission model. The method is applied to fluorescein, FITC and FAM. Depending on the molecule, pH can be measured from pH 1.9 to pH 7.3 with standard deviations between 0.06 and 0.08 pH units. Estimating pH and pKas from shape instead of intensity allows calibration-free measurements even with single-emission peak molecules.

## 1. Introduction

The fluorescence pH dependence of molecules has been studied for decades with the ulterior motive of pH sensing. The literature on pH indicators goes back a long way. However, recent developments in the miniaturization of spectrometers [1] and the current possibilities for advanced and rapid mathematical processing are revitalizing interest in these molecules from the point of view of embedded devices. This will become increasingly important with the development of the IoT. Indeed, embarked signal processing will no longer be necessary, as complicated calculations involving large deported computing facilities can be used. It is therefore worthwhile to take another look at these molecules and assess their new potential in terms of pH measurement.

Describing the hundreds of pH indicators is beyond the scope of this article. Therefore, we will consider two representative molecules for illustration. There are two main classes of pH indicators: those with a single emission peak, such as fluorescein and its derivatives [2,3], and those with two emission peaks, such as SNARF (seminaphtharhodafluor) [4]. For single-emission peak molecules, the pH is calculated by normalizing the emitted intensity at the unknown pH with the intensity recorded at high pH values (high pH for fluorescein and derivatives). Periodic calibration is then required to account for the decrease in indicator concentration due to photobleaching or other reasons. Dual-emission indicators allow ratiometric measurements at emission wavelengths corresponding to the two emission peaks. pH is related to the fluorescence intensity ratio at these two specific wavelengths. The measurement is theoretically calibration-free because the intensity ratio is independent of the indicator concentration. However, the supplier recommends that calibration be performed using solutions of fully protonated and deprotonated forms of the molecule [5]. This aspect will be discussed later. A generalization of ratiometric measurements is used to consider not only intensities at specific wavelengths but also to analyze the whole shape of the emission spectrum ([6], for example). In fact, ratiometric or full-shape pH measurement helps us to move towards calibration-free measurement, since the calculation can be performed on normalized spectra and thus independent of the concentration of the indicator. Note that so-called single-emission peak indicators can be understood as double (or multiple)-emission peak indicators where the emission peaks overlap. Other aspects are the price and the optical properties of pH indicators. Often, double-emission peak molecules are expensive, relatively fragile and have a relatively low quantum yield (about 0.03 for SNARF [7], other indicators in [8]). In contrast, molecules in the fluorescein family are inexpensive, more robust, and have very high quantum yields (about 0.93 [9]).

Therefore, apart from their single-emission peak behavior, fluorescein and its derivatives remain interesting candidates for pH sensing, although some authors have pointed out some disadvantages of using such molecules [10]. For example, it is mentioned that fluorescein has a short lifetime, is prone to photobleaching, and exhibits complex prototropic equilibria that make its spectral fluorescence properties particularly sensitive to pH. In our experience, fluorescein has a much longer lifetime than other dual-emission peak molecules we have tested. It is true that it is prone to photobleaching, but this effect can be greatly reduced by using low excitation intensities. In fact, as long as a fluorescence signal can be detected with an acceptable signal-to-noise ratio, the emission properties of fluorescein can be exploited, especially when using the high-sensitivity spectrometers available today. It is also true that fluorescein and its derivatives exhibit complex prototropic equilibria [11,12]. In fact, this aspect can be exploited to measure pH in a calibration-free manner. Although fluorescein and its derivatives are considered single-emission peak indicators, the shape of this peak evolves slightly with pH. The shape evolution with pH is not as visible as what can be observed with dual-emission peak indicators, but it can be measured and mathematically described as explained in this paper. The idea is that each prototropic form has its own fluorescence emission spectrum. The proportion of prototropic forms present at a given pH is governed by the laws of mass action. It is therefore possible to analyze the shape of the emission peak to deduce the pH value. To do this, the emission shape of each prototropic form must be known, as well as the pKa values. Note that as long as the pKa values are known, measuring the pH from the shape of the emission spectrum is straightforward in cases where there are only two prototropic forms because the shapes of the emission spectra at extreme pH values can be measured directly [6]. It is much more complicated when there are more prototropic forms because it is impossible to isolate the fluorescence contributions of forms present at intermediate pH values.

Depending on its protonated or deprotonated state, fluorescein has seven prototropic (or protolytic, tautomeric according to the authors) forms: one cationic (C), three neutral (Q-quinoid, L-lactone and Z-zwitterion), two monoanionic (MI and MII) and one dianionic (D) [11,12]. For simplicity, these forms are grouped into four classes: cation, neutral, monoanion and dianion. These prototropic forms have been depicted several times (see [12] for pictures. In fact, the three molecules considered in this paper differ only by the addition of an NCS group (FITC) or COOH group (FAM). The protonation and deprotonation of these molecules always follow the same process. The pKas of the ionic transitions depend on the solvent used and the experimental conditions. They range from 2.08 to 2.34 for pKa1, from 4.31 to 4.36 for pKa2, and from 6.43 to 6.84 for pKa3 [3,11,12]. Due to the activity of protons, pKa values depend mainly on the composition of the solution in which the pH indicators are dissolved (ionic strength) and on the temperature. Therefore, accurate measurement of pH requires simultaneous measurement of pKa values.

There is no real consensus on the number of prototropic forms that show fluorescence. Some authors mention that mainly the anion and the dianion are fluorescent with relative intensities of 1 and 0.06 compared to the intensity at pH > 8 [2,11]. The fluorescence of the neutral form is briefly mentioned with a relative intensity of only 0.008. Other authors state that the neutral and cationic forms do not fluoresce but are directly converted to the anionic form upon excitation [3]. It could be concluded that fluorescein can be used for pH sensing only for pH values close to the pKa3 value. However, other publications report studies performed over a wide pH range (pH 0.3 to pH 10.5 [12]) and mention the fluorescence properties of different prototropic forms: dianion, anion and cation [3,13], with the neutral form added in [11,14]. The global fluorescence spectrum of fluorescein at various pH values has been published many times, and examples of spectra recorded over a wide pH range can be found in [12,14]. However, to the best of our knowledge, the shapes of fluorescence spectra of individual prototropic forms have never been published.

In this paper, we propose a simple method to simultaneously measure the shapes of the emission spectra of each prototropic form of fluorescein and some of its derivatives. Taking into account the laws of mass action, complete models of the pH-dependent emission spectra of these molecules in their solvent are constructed. These models are then used to fit experimental spectra to measure the corresponding pH. The potential of these molecules for pH sensing is then discussed. Section 2 of this paper presents the experimental setup used and the methods employed to generate wide ranges of pH values while keeping the ionic strength constant. In Section 3, we present how complete models of the fluorescence emission of these molecules are built and we assess the potential of each of them in terms of pH sensing. A discussion is then proposed in Section 4.

## 2. Experimental Aspects

### 2.1. Experimental Setup

**Figure 1:**
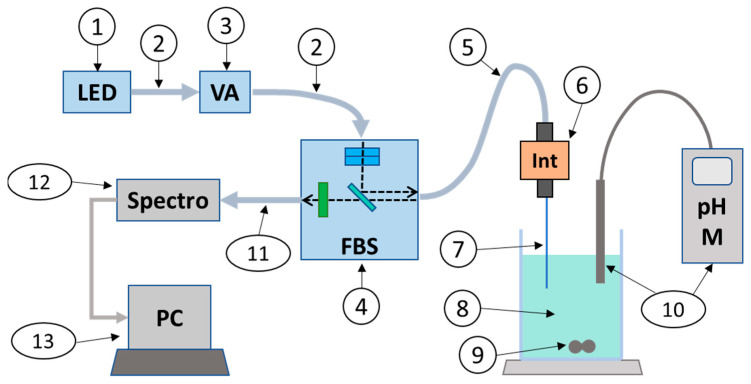
Experimental setup for measuring fluorescence spectra over a wide pH range. Numbers correspond to item numbers in Table S1.

The experimental setup is shown in Figure 1. Details of the elements are given in Table S1 (Supplementary Materials).

The excitation light was emitted from an LED with a center wavelength of 470 nm (part 1). It was launched into a multimode fiber (200 µm core, NA 0.22) (part 2), passed through a variable attenuator (part 3), and injected into a fluorescence beam splitter (FBS) (part 4) via another multimode fiber. The FBS was equipped with micro-lenses, 2 excitation filters to narrow the excitation spectrum and improve rejection of the excitation wavelength in the emission region, and an emission filter coupled to a dichroic mirror to isolate the fluorescence signal above 490 nm. The excitation bandwidth was 25 nm centered at 470 nm. We chose not to center the excitation on the absorption maximum (488 nm) to better reject the excitation wavelengths from the emission spectrum.

The excitation light was coupled to a custom-fabricated multimode patch cable (100 µm, NA 0.22) (part 5) terminated with a 2.5 mm diameter ferrule inserted into a quick-release connector. A custom-fabricated, ferruled, bare and cleaved fiber (200 µm, NA 0.22) (part 7) allowed the fluorescence to be excited in the test solution (part 8). The solution was continuously homogenized with a magnetic stirrer (part 9) and the pH was continuously measured with a commercial pH meter (part 10).

The induced fluorescence propagated back to the FBS (between 490 nm and 830 nm wavelength) through a multimode fiber (400 µm, NA 0.22) (part 11) to a high-sensitivity spectrometer (wavelength range 348–1127 nm, resolution 1 nm) (part 12). Spectra recorded at each pH were then stored in a PC (part 13).

### 2.2. pH Controlable Solution

The test solution consisted of 1 M NaCl solution to which fluorescein (or derivative) was added. The fluorescence spectrum was continuously recorded and fluorescein was added until the fluorescence signal-to-noise ratio (SNR) reached approximately 500. The exact amount of fluorescein was not important as only the shape of the fluorescence spectra was of interest. The pH was then neutral and the ionic strength was 1 M. To change the pH of the solution while keeping the ionic strength constant, two ion exchange strategies were used.

A first series of experiments was performed from neutral to alkaline pH values. OH^−^ ion exchange resin was used to increase the pH. This resin absorbs Cl^−^ ions and releases OH^−^ ions into the test solution, thus increasing the pH while keeping the ionic strength constant. Absorption of anionic forms of fluorescein and its derivatives by the resin was not observed. Starting from neutral pH, increasing amounts of resin were added to create a pH range. After each resin addition, the pH was measured with a commercial pH meter and a fluorescence spectrum was measured.

A second series of experiments was performed from neutral to acidic pH values. Nafion was used to lower the pH. Nafion is a nano-porous polymer with internal surfaces coated with sulfonate ions. These interact with the protons of the water that fills the nano-pores. The affinity of Nafion is greater for Na^+^ than for protons. When immersed in NaCl solution, Nafion absorbs Na^+^ and releases H^+^ into the test solution, lowering the pH while keeping the ionic strength constant. Starting from neutral pH, increasing amounts of Nafion were added to create a pH range. After each addition of Nafion, the pH was measured with a commercially available pH meter and a fluorescence spectrum was measured. However, Nafion has the disadvantage of also having a greater affinity for the cationic form of fluorescein and its derivatives than for H^+^. Consequently, at pH 4 and below, the fluorescein concentration decreased with pH and the SNR of the recorded spectrum decreased. In addition, the absorption of fluorescein decreases with pH, further degrading the SNR [15]. To maintain a sufficiently high SNR, fluorescein was periodically added to the test solution while the pH was lowered. The pH ranges and the number of pH values for each tested molecule are given in Table 1.

Spectra were recorded at room temperature (22 °C), and the pH meter was recalibrated periodically, especially for low and high pH values.

### 2.3. Additional Signal and Fluorescence Spectra Acquisition

Spectra were acquired using Oceanview Spectroscopy software, version 2.0.15, from Ocean Optics, USA (French supplier, IDIL Fibres Optiques, Paris, France). The integration time was 10 s with no boxcar or multispectral averaging. For each measurement, a dark background (LED off) was acquired and subsequently subtracted from the recorded spectrum (LED on).

Despite the use of the fluorescence beam splitter, which includes 2 excitation filters, 1 emission filter and a dichroic mirror, a portion of the LED spectrum above 490 nm was still detected by the spectrometer. In addition, a non-negligible spurious fluorescence signal was generated in the optical fibers (contribution of the buffer, epoxy glue in the connectors…) and possibly also in the FBS. This represented an additional fluorescence signal that was removed from each recorded spectrum. To do this, this additional signal was recorded before the addition of fluorescein in the 1 M NaCl solution. It was then subtracted from each recorded spectrum prior to subsequent mathematical processing. The spectra were then normalized.

Numerical developments were performed using MATLAB^TM^ (R2020b version from MathWorks, Natick, MA, USA). Parameters used to describe the shapes of the prototropic forms of the molecules were calculated using MATLAB^TM^’s “fminsearch” minimization function for wavelengths above 510 nm. Once the models were established, the pH was fitted from experimental spectra using the NonlinearLeastSquares algorithm of the MATLAB^TM^ Curve Fitting toolbox for wavelengths above 510 nm with initial test pH values between 0 and 16 and a starting point of 8.

## 3. Results

In this section, we first establish the mathematical expression describing the emission spectrum of fluorescein and its derivatives as a function of pH. In fact, the equation we obtained is valid for any fluorescent molecule that has four classes of prototropic forms. We assume that the three neutral forms contribute to the shape of the “neutral” form mentioned below. The same is true for the anionic forms. We then fit experimental spectra measured with the three fluorescein derivatives considered in this study. Theoretical models of their fluorescence emission vs. pH are then established. Finally, we used these results to evaluate the potential of these molecules as fluorescence pH sensors.

### 3.1. Modeling the Fluorescence Spectra of Fluorescein and Derivatives

The shapes of the fluorescence spectra depend on the proportions of each prototropic form present at a given pH and on the shapes of their corresponding emission spectra. The proportions of each prototropic form can be found, for example, in [10]. They are written as follows:

(1) $$F_{c}(pH,\;p\tilde{K}a_{i})=\left[Cation\right]/\left[Fluorophore\right]=\frac{1}{1+\frac{{\tilde{K}a}_{1}}{\left[H^{+}\right]}+\frac{{\tilde{K}a}_{1}{\tilde{K}a}_{2}}{{\left[H^{+}\right]}^{2}}+\frac{{\tilde{K}a}_{1}{\tilde{K}a}_{2}{\tilde{K}a}_{3}}{{\left[H^{+}\right]}^{3}}}$$

(2) $$F_{n}(pH,\;p\tilde{K}a_{i})=\left[Neutral\right]/\left[Fluorophore\right]=\frac{1}{\frac{\left[H^{+}\right]}{{\tilde{K}a}_{1}}+1+\frac{{\tilde{K}a}_{2}}{\left[H^{+}\right]}+\frac{{\tilde{K}a}_{2}{\tilde{K}a}_{3}}{{\left[H^{+}\right]}^{2}}}$$

(3) $$F_{a}(pH,\;p\tilde{K}a_{i})=\left[Anion\right]/\left[Fluorophore\right]=\frac{1}{\frac{{\left[H^{+}\right]}^{2}}{{\tilde{K}a}_{1}{\tilde{K}a}_{2}}+\frac{\left[H^{+}\right]}{{\tilde{K}a}_{2}}+1+\frac{{\tilde{K}a}_{3}}{\left[H^{+}\right]}}$$

(4) $$F_{d}(pH,\;p\tilde{K}a_{i})=\left[Dianion\right]/\left[Fluorophore\right]=\frac{1}{\frac{{\left[H^{+}\right]}^{3}}{{\tilde{K}a}_{1}{\tilde{K}a}_{2}{\tilde{K}a}_{3}}+\frac{{\left[H^{+}\right]}^{2}}{{\tilde{K}a}_{2}{\tilde{K}a}_{3}}+\frac{\left[H^{+}\right]}{{\tilde{K}a}_{3}}+1}$$

In these equations, $\left[Fluorophore\right]$ is the total fluorophore concentration, [*Cation*], [*Neutral*], [*Anion*] and [*Dianion*] are the concentration of cationic, neutral, anionic and dianionic forms, respectively, $\left[H^{+}\right]$ is the proton concentration and ${\tilde{K}a}_{i}$ is what we call an “adjusted” association constant (see Section 4 for the signification of ${\tilde{K}a}_{i}$ and $p\tilde{K}a_{i}$).

We assume that the four prototropic shapes produce fluorescence. In spectroscopy, spectra are usually decomposed into a sum of Gaussian functions. Transposed to the wavelength domain used in optics, these Gaussian functions are written as follows:

(5) $$G^{WT}\left(\lambda \right)=A\;exp\left[-{\left(10^{7}\frac{\left(\frac{1}{\lambda }-\frac{1}{Y}\right)}{T}\right)}^{2}\cdot 4\log\left(2\right)\right]$$

In Equation (5), A is the amplitude (in arbitrary units), Y is the position of the Gaussian (in nm) and T is the width of the Gaussian (in cm^−1^).

We found that three Gaussian functions efficiently describe the shape of the emission of each prototropic form. Each prototropic form is written as follows, where the letters ‘c’, ‘n’, ‘a’ and ‘d’ refer to cation, neutral, anion and dianion, respectively.

(6) $$cation\left(\lambda \right)=\sum_{i=1}^{3}Aci\;exp\left[-{\left(10^{7}\frac{\left(\frac{1}{\lambda }-\frac{1}{Yci}\right)}{Tci}\right)}^{2}\cdot 4\log\left(2\right)\right]$$

(7) $$neutral\left(\lambda \right)=\sum_{i=1}^{3}Ani\;exp\left[-{\left(10^{7}\frac{\left(\frac{1}{\lambda }-\frac{1}{Yni}\right)}{Tni}\right)}^{2}\cdot 4\log\left(2\right)\right]$$

(8) $$anion\left(\lambda \right)=\sum_{i=1}^{3}Aai\;exp\left[-{\left(10^{7}\frac{\left(\frac{1}{\lambda }-\frac{1}{Yai}\right)}{Tai}\right)}^{2}\cdot 4\log\left(2\right)\right]$$

(9) $$dianion\left(\lambda \right)=\sum_{i=1}^{3}Adi\;exp\left[-{\left(10^{7}\frac{\left(\frac{1}{\lambda }-\frac{1}{Ydi}\right)}{Tdi}\right)}^{2}\cdot 4\log\left(2\right)\right]$$

Finally, the complete emission spectrum can be written as follows:

(10) $$\begin{array}{cc} Spectrum\left(pH,\;p\tilde{K}ai,\;\lambda \right)= & F_{c}\left(pH,\;p\tilde{K}a_{i}\right)\cdot cation\left(\lambda \right)+F_{n}\left(pH,\;p\tilde{K}a_{i}\right)\cdot neutral\left(\lambda \right)+\\ & F_{a}\left(pH,\;p\tilde{K}a_{i}\right)\cdot anion\left(\lambda \right)+F_{d}\left(pH,\;p\tilde{K}a_{i}\right)\cdot dianion\left(\lambda \right) \end{array}$$

In total, 39 parameters must be calculated for each type of fluorescein and its derivatives: 3 “adjusted” association constants and 36 shape parameters.

### 3.2. Experimental and Mathematical Measurement of Spectra Parameters

**Figure 2:**
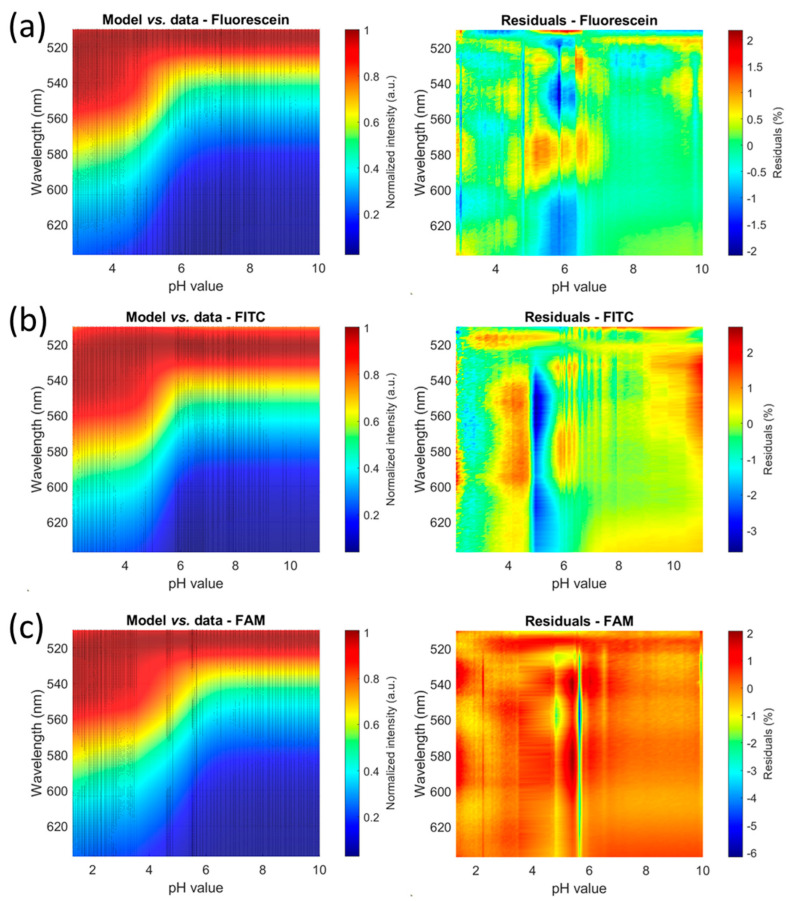
Comparison between experimental spectra and numerical models measured with fluorescein and its derivatives (excitation wavelength 25 nm span centered at 570 nm). View with 90° elevation. Left figures: normalized spectra, semi-transparent colored surface: model, black dot: experimental data. Right figures: residuals expressed in percentages. (**a**) Fluorescein. (**b**) FITC. (**c**) FAM.

Experimental spectra were then measured as described in Section 2, starting from 1 M NaCl (i.e., 1 M ionic strength). Additional signal was removed and spectra were normalized. Figure 2 shows spectra recorded with fluorescein (a), FITC (b) and FAM (c). Experimental spectra and calculated models are shown in the left column. Black dots are experimental values, and semi-transparent colored shapes represent the calculated models. The right column represents the residuals expressed in % (see Section 4.2 for the different aspects of residuals of FAM).

Parameters are estimated using the fminsearch function in MATLAB^TM^. The method consists of finding the set of parameters that minimize the following error function.

(11) $$error=\sum_{\lambda }\sum_{pH}{\left(Spectrum\left(pH,\;p\tilde{K}ai,\;\lambda \right)-ExpSpectra(pH,\;\lambda )\right)}^{2}$$

In this equation, $Spectrum\left(pH,\;p\tilde{K}ai,\;\lambda \right)$ is given by Equation (10), and ExpSpectra(pH, λ) are the experimental normalized spectra.

This function requires the definition of initial values for each parameter to be optimized. Finding these starting points is quite empirical and time-consuming. There are not many criteria to guide the choices, except that the spectral amplitudes of each prototropic form should ideally be equal to 1. However, starting points concerning the Gaussian function describing the shape of the prototropic forms were approximately determined using a few fittings of fluorescein spectra at pH values covering the whole pH range. Starting values for $\tilde{K}a_{i}$ were arbitrarily set to 10^−2^, 10^−5^ and 10^−6^, respectively, corresponding to $p\tilde{K}a_{i}$ values of 2, 5 and 6. Table S2 (Supplementary Materials) shows the parameters calculated for each molecule, as well as the values of the starting points (which are the same for each molecule). Note that the minimization algorithm does not provide uncertainties. The values of the corresponding $p\tilde{K}a_{i}$ of the three species are given in Table 2.

### 3.3. Fractions and Fluorescence Emission Shapes of Prototropic Forms

**Figure 3:**
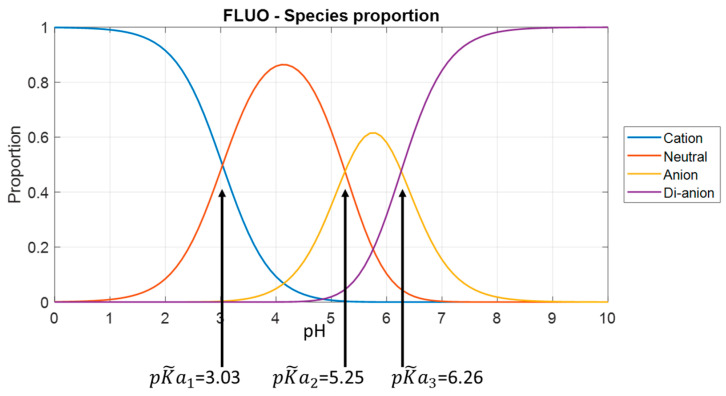
Species proportions of fluorescein in a 1 M NaCl solution.

**Figure 4:**
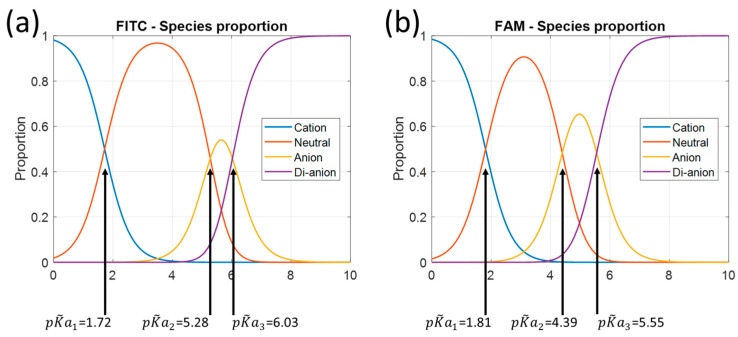
Species proportions of FITC (**a**) and FAM (**b**) in a 1 M NaCl solution.

Figure 3 shows the fraction of prototropic forms of fluorescein versus pH in the 1 M ionic strength test solution. Figure 4 shows the fractions for the other molecules. These figures are plotted using Equations (1)–(4) with the adjusted association constant values from Table 2.

Referring to Table 1, we obviously lack data for low pH values, mainly for fluorescein and FITC. In fact, for these two molecules, the lowest pH tested is approximately equal to $p\tilde{K}a1$. At this pH, the solution contains equal amounts of cationic and neutral forms. It is likely that the description of the cationic form can be improved. This is much less the case for FAM, where the fractions are 80% and 20% for the cationic and neutral forms, respectively. At high pH, there is no problem.

**Figure 5:**
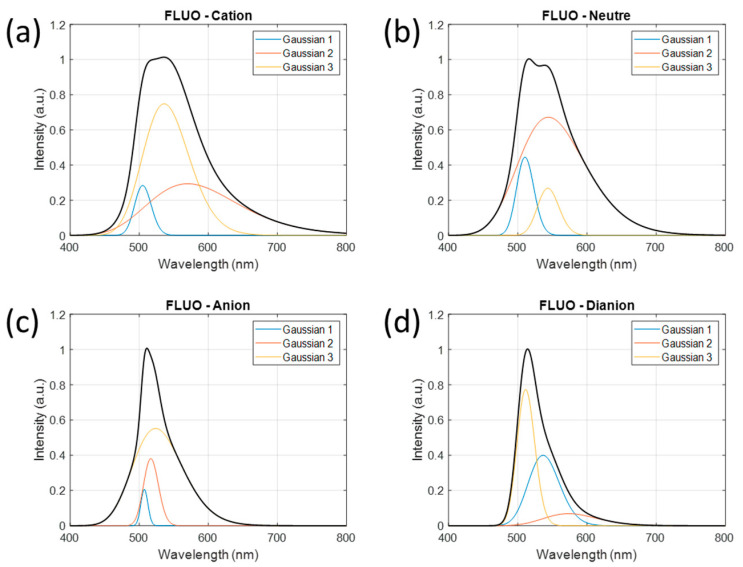
Emission spectrum shapes of prototropic forms of fluorescein in 1 M NaCl solution. Spectrum shapes of each form are described using 3 Gaussian functions. (**a**) cation form, (**b**) neutral form, (**c**) anionic form and (**d**) di-anionic form.

The emission spectrum shapes of the prototropic forms of fluorescein are shown in Figure 5 along with the three “wavelength transposed” Gaussian decomposition of each form. Note that the amplitude of the cation form is slightly greater than 1. This may be due to the lack of data at low pH values (see Table 1). This is discussed in Section 4.

### 3.4. Fitting pH from Experimental Spectra

**Figure 6:**
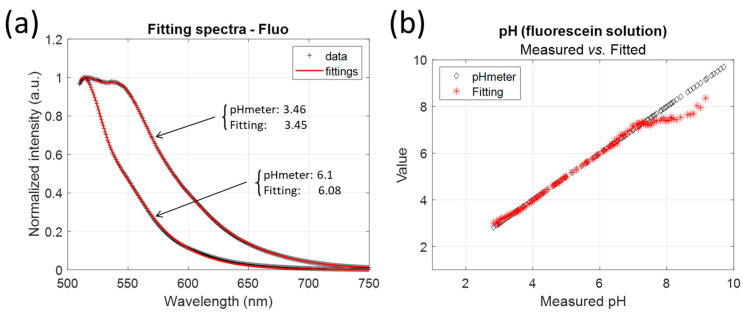
Examples of pH determination by fitting experimental spectra to the mathematical model. Measurements with fluorescein. (**a**) Examples at pH 3.46 and pH 6.1. (**b**) Comparison of “calculated” vs. “measured” over the whole range.

Knowing the parameter values and the $p\tilde{K}ai$ makes it possible to build a complete model of the fluorescence emission of each molecule with pH. Figure 6a shows examples of experimental spectra fitting and the values of the calculated pH compared to the experimental pH values. In these examples, the error in the pH value is 0.01 and 0.02 pH units for pH values of 3.46 and 6.1, respectively. Fitting is very accurate with R2 values always greater than 0.999 (the same for FITC and FAM). However, the pH measurement range is reduced, as shown in Figure 6b. The model is unable to fit the correct pH value for pH above 7.3 for fluorescein.

**Figure 7:**
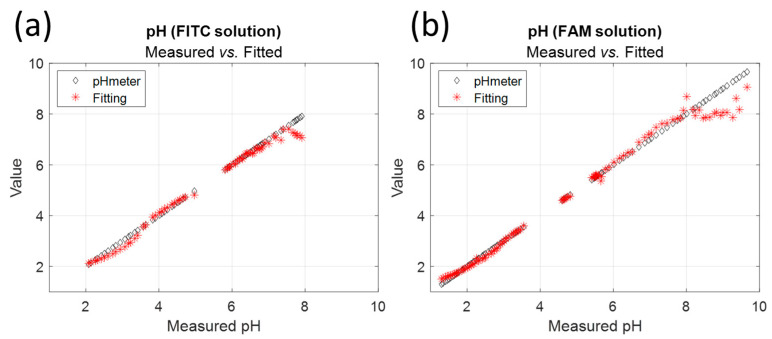
pH fitting vs. measurement with pH meter for (**a**) FITC and (**b**) FAM.

Figure 7 shows the pH values calculated by fitting compared to the values measured with the pH meter for FITC and FAM. In all cases, the model fails to give the correct pH for high pH values. In fact, as can be seen in Figure 2b, the shape of the emission spectrum remains almost constant above pH 7.3, which prohibits its use for pH sensing. This is further confirmed by the evolution of the fraction shown in Figure 4.

The measurement range is reduced for each molecule. This aspect is discussed in Section 4.

Within the measurement range, fitting produces pH values with an accuracy of less than 0.01 pH units for fluorescein and 0.02 pH units for FITC and FAM. However, this represents the uncertainty of fitting and the actual error made during pH determination. An estimation of the pH determination accuracy is given by the standard deviation of the difference between the pH meter-measured pH and the calculated pH. The ranges and standard deviations for each molecule are summarized in Table 3. Fluorescein has the smallest standard deviation but over a smaller range than FAM. FITC appears to be the least suitable candidate for pH sensing. However, the pH range can potentially be extended to low pH values if measurements are made in this range and the model is built accordingly.

## 4. Discussion

### 4.1. Concerning pH Measurements

In solution, fluorescein exists in both the open-loop (fluorescent) and spirocyclic (non-fluorescent) forms. Since the solutions fluoresce, some or all of the molecules are in the spirocyclic form. Our goal is to measure pH from the shape of the fluorescence spectra, not to measure fluorescein concentration. Therefore, it is valid to consider only the open forms to model the pH behavior of these molecules.

Fluorescent molecules suffer from photobleaching, which can alter the fluorescence spectrum during spectral acquisition. At least, this is true if the spectrometer used relies on the use of a tunable monochromator. In our case, the spectrometer uses a diffraction grating and a CCD linear array. The entire spectrum is therefore acquired without scanning. If photobleaching does occur, this is reflected in a drop in the intensity detected during the acquisition period but no change in the shape of the acquired spectrum. Furthermore, measurements were carried out in solution, and only molecules present in the fiber-optic illumination cone were subject to photobleaching. At each pH change, the solution is homogenized by magnetic stirring, which renews the excited molecules that have not yet undergone photobleaching. During the measurement campaigns, we did not notice any photobleaching sufficient to jeopardize the acquisitions.

We evaluated the potential of the tested molecule in terms of pH sensing by fitting experimental spectra to the models we had built. In other words, we tested a model with the data that were used to build it, which is somewhat unfair. The correct method would have been to consider cross-validations [16]. However, the goal of this paper is not to build a pH sensor and measure its performance. In Section 3.4, we simply evaluated the potential of the tested molecules for pH sensing.

It is likely that the pH-sensing range can be increased, especially in the acidic region. Indeed, it can be observed in Figure 2 that the shapes of the spectra continue to evolve at pH values lower than those we tested. This was not anticipated in the design of the experiments. We used a 1 M ionic strength NaCl solution in order to be able to change the pH relatively quickly by ion exchange without changing the ionic strength. Table 2 shows that the pKa_s_ we estimated are not lower than those reported in the literature for lower ionic strengths. Consequently, the shape of the emission spectrum is modified at lower pH values than expected. Modification of pKa values with ionic strength is not straightforward. pKa_s_ can either increase or decrease with ionic strength depending on the buffer in which the indicator is dissolved [17]. Further experiments should now be performed, including measurements at much lower pH values. This will better define the emission shape of the cationic forms and probably extend the pH range for low pH. It is unlikely that the high pH range can be extended. In fact, Figure 2, Figure 3 and Figure 4 show that the shape of the spectrum remains constant at high pH values because only dianions are present.

Looking at Figure 3 and Figure 4 and Table 3, it appears that the models fail at high pH when the dianion fraction reaches about 0.9. This is true for every molecule tested. The models do not work from this point onwards, as the shapes of the spectra hardly change at all. On the other hand, we have no explanation for the fact that this happens at pH 7 for fluorescein and FITC and pH 6.5 for FAM. Conversely, at low pH, the model is expected to remain valid until the cation fraction reaches 0.6 for both fluorescein and FAM. FITC behaves differently. Considering this limit of the 0.6 cation fraction, the model should be valid down to pH 2, but the lower limit of the range was 3.5. This is not explained yet. This relatively high lower pH detection limit is mainly due to the poor accuracy with which the shape of the cationic form can be estimated (the tested pH is not low enough). The upper measurement range limit seems to coincide with a dianion fraction of 0.9. If we consider the same rule for the lower limit, pH could potentially be measured until the cation fraction reaches the same value. This means that potentially, lower limits could be pH 2 for fluorescein and 1 for both FITC and FAM. The lower value for FITC and FAM is due to the reduced pKa1 for these molecules. The potential pH measurement range (about 6 pH units) is then much larger than the 2 pH unit range commonly mentioned in the literature ([18], for example).

Another question arises: what is the minimum measurable pH change? The STD values given in Table 3 assess the accuracy with which pH can be measured using the current model, based on currently recorded spectra. They do not represent the minimum measurable pH variation. To assess this, we should consider that the spectra used to build the model are perfectly recorded and that the pH measurements made with the pH meter are perfect. In this case, the minimum measurable pH variation would be related to the uncertainty with which the spectra can be fitted using Equation (10), i.e., 2 × STD (fitting uncertainty). Theoretically, we obtain ΔpH = 0.009 pH units for fluorescein, ΔpH = 0.014 pH units for FITC and ΔpH = 0.011 pH units for FAM (to be compared with the values in Table 3).

### 4.2. Concerning the Mathematical Model and Ways to Simplify/Improve It

Currently, the model requires 39 parameters to be determined. The minimization algorithm needs starting points for these parameters. They were determined approximately as explained in Section 3.2. The residuals shown in Figure 2 represent the differences between the model and the experimental data. The residuals are in the range of ±1% for fluorescein, ±2% for FITC and ±1% for FAM, demonstrating the accuracy of the calculated models. Note that the residuals of FAM look different from the others. This is because the color bar is shifted to the negative values due to the spectrum recorded at pH 5.66, which is largely higher than the model in the 440–480 nm wavelength range. The STD values presented in Table 3 show that the fit of the experimental data with the calculated model is robust.

This was not carried out in this work, but the number of parameters can be greatly reduced. In fact, the prototropic form parameters for extreme pH values can be measured directly because only one prototropic form exists in these pH ranges. The number of parameters required would then be reduced to 21. In the work presented here, the pH tested was not low enough to isolate the cationic forms. However, we could have reduced the number of parameters to 30. The model would have been simplified, but it is not certain that its accuracy would have been greatly improved.

An additional simplification was suggested by one of our reviewers: “The fitting may be further improved by estimating the amplitude parameters with a least-squares decomposition into the Gaussian shapes within the residual function. Each individual spectrum would therefore only require 6 fit parameters as the 3 amplitudes are estimated implicitly”. Another suggestion was “Instead of modeling the fluorescence spectra by multiple Gaussians, the spectra might be determined with a least-squares decomposition or a parallel factor analysis” [19,20,21]. We will consider these suggestions in future work.

### 4.3. Concerning Other Applications of the Modeling Method

The model proposed in this paper allows us to define the shape of the fluorescence emission spectra of the prototropic forms of studied molecules. As mentioned in the previous section, forms existing at extreme pH values can be measured directly as only cationic or dianionic forms exist. This is not the case for neutral or anionic forms as they exist together with other forms. The only way to determine them is to build the global model proposed in this paper. This method can be used for other purposes.

#### 4.3.1. Application 1: Evaluating the Quantum Yields of Different Prototropic Forms

Working on non-normalized spectra could enable us to estimate the relative emissions of each form. To do this, we would need to make sure that we are working with constant concentrations of fluorescent molecules and that these are not subject to photobleaching.

However, we used Nafion to lower the pH, which decreased the concentration of fluorescein in the test solution. For this reason, quantities of fluorescent molecules were added regularly. We therefore have no real measurement of the intensity emitted at a fixed concentration. The same question can be asked at alkaline pH with OH- resin, although we did not observe any change in resin color, as was the case with Nafion (see Section 4.6). The manipulations carried out were only intended to measure the shape of the spectra, so as to progress towards a calibration-free measurement and not to measure the quantum efficiencies of each of the forms.

#### 4.3.2. Application 2: Determination of Shapes of Absorption Spectra of Prototropic Shapes

The method of modeling fluorescence spectra presented here can also be used to measure the absorption spectra of the different prototropic forms. Equation (10) remains valid, but the parameters describing the shapes of the different forms change (Equations (6)–(9)). To determine them, we need to repeat the modeling method with absorption spectra instead of fluorescence spectra, by illuminating the test solutions with a white light source. This application is only valid if the open-loop and spirocyclic forms are assumed to have the same absorption spectra.

### 4.4. Concerning the Adjusted Association Constants and Proton Activity

As mentioned above, pKa values depend on the temperature and the ionic strength of the solution. In fact, prototropic equilibria also depend on the proton activity, which depends not only on these two factors, as we will see below. Several theories allow the calculation of activity coefficients as a function of ionic strength, ion valence, ion-specific parameters, and parameters dependent on temperature and dielectric constant [22]. It seems illusory to consider all these aspects in a general model. We therefore decided not to modify Equations (1)–(4), which are generally used for a proton activity coefficient equal to 1 (low ionic strength, low ion concentrations), but to include them in a so-called adjusted association constant.

In all cases, using fluorescence, pH cannot be measured alone. Adjusted association constants must be measured simultaneously as they depend on the immediate environment of the pH indicator. This is the main reason why calibrations are required (not only to account for varying indicator concentrations, e.g., due to photobleaching). Originally, some pH indicators were developed for the assessment of intracellular pH by observation with a fluorescence microscope [23,24]. The accuracy of such measurements is questionable, but the goal was visual observation and probably not accurate pH measurement. However, a study presenting ratiometric imaging has been proposed to partially solve the problem of simultaneous pH and pKa measurements [25].

### 4.5. Concerning Simultaneous pH and pKa Measurements and Calibration-Free Measurements

The mathematical description of fluorescence emission is not new. For example, the emission spectrum of SNARF has been described by mathematically defining the spectral shapes of the fully protonated and deprotonated forms [6]. The shape of a spectrum recorded at an unknown pH is analyzed by the fractions of the two prototropic forms of SNARF. This is a generalization of ratiometric measurements to the whole emitted spectrum. For indicators that exist in only two prototropic forms, the fractions of each form are written as follows:

(12) $$\frac{\left[A^{-}\right]}{\left[A\right]}=\frac{1}{1+\frac{\left[H^{+}\right]}{Ka}}=\frac{1}{1+10^{\left(pKa-pH\right)}}$$

(13) $$\frac{\left[AH\right]}{\left[A\right]}=\frac{1}{1+\frac{Ka}{\left[H^{+}\right]}}=\frac{1}{1+10^{\left(pH-pKa\right)}}$$

It is obvious that for indicators with only two prototropic forms, the simultaneous determination of pH and pKa is impossible because there are two equations but only one unknown: either [H^+^/Ka] or (pKa − pH). Therefore, calibrations are required to estimate the value of the pKa using different titration calibration solutions. An equation expressing the value of the pKa is written below (see [5] for an explanation of the parameters in Equation (14)).

(14) $$pH=pK_{a}-{log}_{10}\left[\frac{R-R_{B}}{R_{A}-R}\times \frac{F_{B(\lambda 2)}}{F_{A(\lambda 2)}}\right]$$

Calibration-free measurements require indicators with more than two prototropic forms in order to decorrelate pH and pKa_s_. This is the case for fluorescein, as shown in Equations (1)–(4). Another condition is that at least two of these prototropic forms show fluorescence. However, even for indicators with multiple prototropic forms, calibration-free pH measurement is not possible without measuring pKa_s_, since pKa_s_ are dependent on the solvent and the experimental conditions.

### 4.6. Potential Calibration-Free Fluorescence pH Measurement with Grafted Molecules

In this study, we worked with a very high ionic strength (1 M). One possibility, for lower ionic strengths, is to introduce into the system an element that we can control perfectly and that has a much greater influence on proton activity than the composition of the solution and/or the experimental conditions. This additional element will act on the pKa_s_ in such a way that the other effects become negligible.

**Figure 8:**
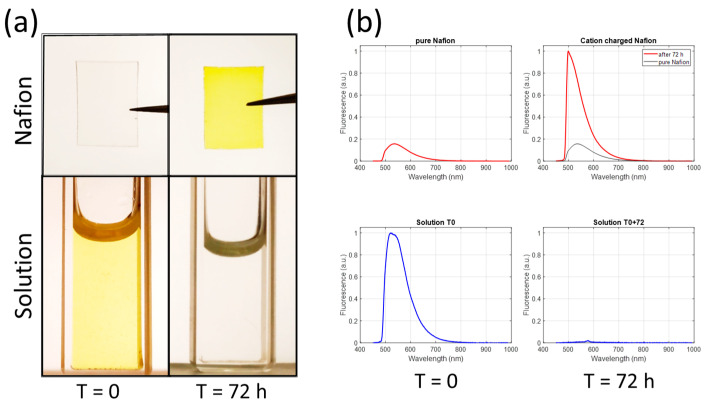
Effect of surface on fluorescence emission of fluorescein. (**a**) Pictures of Nafion and solution at pH 0.47 at T = 0 and T = 72 h. (**b**) Emission spectra of solution and Nafion at T = 0 and T = 72 h.

The idea is to graft or deposit the molecules onto a surface. In fact, surfaces have an important effect on the ionic activities. The range of accessible pH (i.e., the effective pKa) can shift by 3 pH units towards lower values, although the shift can be positive in some cases [18]. pH shifts have been observed experimentally and studied theoretically with SNARF contained in nanochannels by considering the ζ-potential [26]. We have also observed drastic effects of the surface on the emission properties of fluorescein. We recall that Nafion was used to exchange Na^+^ ions with protons contained in the nanopores of Nafion. We also observed that at low pH, the cationic form of fluorescein was exchanged with protons. We measured the fluorescence emission of a pH 0.47 solution and Nafion before and after immersion of the Nafion in the solution (Figure 8).

First, the migration of cations into the Nafion is clearly visible in Figure 8a. Second, a very large shift in the emission spectra of cation-charged Nafion is observed in Figure 8b. This demonstrates the enormous effect of the surface on the emission characteristics. Of course, in this example, the effect of the surface is impressive because the electrostatic interaction between cations and sulfonate ions is extremely strong. This also shows that when grafted (or adsorbed) onto a surface, the proton activity is strongly modified, which is consistent with the above hypothesis of calibration-free possibilities.

Fluorescein or derivatives have been attached to the end of optical fibers for pH sensing. For example, FITC was used in [27] for relative intensity measurements and in [28] for direct intensity measurements. However, no mention was made of a possible calibration-free measurement. In light of the above, there are a number of avenues to explore in order to move towards calibration-free measurements. First, molecules must be covalently grafted, which can be carried out using silanization [29]. The SNR must be high enough to allow the construction of an accurate model. One possibility is to grow a very thick aminosilane layer onto which fluorescein molecules are grafted. However, such thick layers make it difficult to obtain reproducible layers and the establishment of an accurate model. Double silanization is an option [30]. The indicator layer must then be as reproducible as possible, which can be achieved using the method described in [31]. In this way, it is likely that a fluorescein-based calibration-free pH sensor can be fabricated using the spectroscopic data processing described in this paper. Obviously, the use of such sensors would be calibration-free, but an initial calibration should be performed during sensor fabrication to establish a mathematical model.

## 5. Conclusions

This paper reports, for the first time to our knowledge, a simple method for mathematically describing the shape of the fluorescence spectrum of any prototropic form of fluorescein and some of its derivatives. This method can be applied to any fluorescent pH indicator. In our study, each prototropic form is described using three Gaussians, but the number of Gaussians can be adjusted as needed. The use of Gaussians is not mandatory; any other suitable function can be used.

A mathematical model of the fluorescence emission as a function of pH is then constructed. Using this model and depending on the molecule, pH can be measured in the range of 1.9 to 7.3 pH units with standard deviations between 0.06 and 0.08 pH units. The range can potentially be extended to pH 1.

Using this mathematical description and considering sensor architectures that allow the control of indicator layer structures that have a strong effect on ion activity, calibration-free fluorescence pH sensors can be envisioned.

## Figures and Tables

**Table 1:** pH range and number of pH values per molecule.

Fluorophore	Min. pH	Max. pH	Sample
Fluorescein	2.83	9.7	108
FITC	2.08	7.91	64
FAM	1.3	9.66	94

**Table 2:** Values of the “adjusted” $p\tilde{K}a_{i}$ of each molecule.

		Molecule	
	Fluorescein	FITC	FAM
$p\tilde{K}a_{1}$	3.03	1.72	1.81
$p\tilde{K}a_{2}$	5.25	5.28	4.39
$p\tilde{K}a_{3}$	6.26	6.03	5.55

**Table 3:** pH measurement ranges and standard deviations for each molecule.

Molecule	pH Min	pH Max	STD
Fluorescein	3.5	7.3	0.06
FITC	3.5	7	0.08
FAM	1.9	6.5	0.08

## Data Availability

Research data are available on demand to the corresponding author.

## Supplementary Materials

The following supporting information can be downloaded at https://www.mdpi.com/article/10.3390/s24051705/s1. Table S1: Designation, supplier, and part numbers. Table S2: List of parameters for each molecule including starting values for the minimization algorithm.

## Abbreviations

Fluo	Fluorescein
FITC	Fluorescein 5-isothiocyanate
FAM	6-Carboxyfluorescein
SNR	Signal-to-noise ratio
