# Supplementary material for: Fluorescence Spectra of Prototropic Forms of Fluorescein and Some Derivatives and Their Potential Use for Calibration-Free pH Sensing
Source: Sensors (Basel). 2024 Mar 6;24(5):1705. doi: 10.3390/s24051705 (PMC10934543; doi:10.3390/s24051705)
Supplement: Supplementary file 1 [file sensors-24-01705-s001.zip › sensors-2866059-supplementary.pdf]

sensors MDPI

*Supplementary Materials for*

# Fluorescence Spectra of Prototropic Forms of Fluorescein and Some Derivatives and Their Potential Use for Calibration-Free pH Sensing

**Bernard Gauthier-Manuel [1], Chafia Benmouhoub [1,2] and Bruno Wacogne [1,3,*]**

[1] CNRS, Institut FEMTO-ST, Université de Franche-Comté, 25000 Besançon, France; bernardgauthier55@gmail.com (B.G.-M.); benmouhoubc@gmail.com (C.B.)
[2] SATT Grand Est, SAYENS, 25000 Besançon, France
[3] INSERM CIC 1431, Besançon University Hospital, 25030 Besançon, France
* Correspondence: bruno.wacogne@univ-fcomte.fr; Tel.: +33-3-81-66-63-88

**Table S1.** Designation, supplier, and part numbers.

| Part N° in Figure 1 | Designation | Supplier/Country | Reference |
|---|---|---|---|
| 1 | Light emitting diode | Thorlabs, USA, supplier France | M470F3 |
| 2 | Multimode patch cable | Thorlabs, USA, supplier France | M19L01 |
| 3 | Variable attenuator | CivilLaser, China | FVA-UV |
| 4 | Fluorescence beam splitter<br>Including:<br>Exc. filter #1<br>Exc. filter #2<br>Dicroic mirror<br>Em. filter | DORIC Lenses, Canada<br><br>Semrock, supplier France Optoprim<br>Semrock, supplier France Optoprim<br>Semrock, supplier France Optoprim<br>Semrock, supplier France Optoprim | Custom assembled<br><br>FF01-457/50-25<br>FF01-474/27-25<br>LPD02-488RU-25<br>LP02-488RU-25 |
| 5 | Custom patch cable | DORIC Lenses, Canada | Custom assembled |
| 6 | Ferule interconnect | Thorlabs, USA, supplier France | ADAF2 |
| 7 | Custom bare fiber<br>Including:<br>Multimode fiber | DORIC Lenses, Canada<br><br>Thorlabs, USA, supplier France | CuFG200UEAstom assembled |
| 8 | Test solution<br>Containing:<br>NaCl 1M<br><br>Fluorescein<br><br>FITC<br>FAM<br>pH adjusted with:<br>Nafion, 50 µm<br>OH- ion exchange resin | <br><br>Merck, Sigma Aldrich, Germany, supplier France<br><br>Merck, Sigma Aldrich, Germany, supplier France<br><br>MedChemTronica, Sweden<br>MedChemTronica, Sweden<br><br>IonPower, US<br>Merck, Sigma Aldrich, Germany, supplier France | <br><br>CAS: 7647-14-5<br><br>CAS: 2321-07-5<br><br>CAS: 3326-32-7<br>CAS: 3301-79-9<br><br>NR212<br>CAS: 39339-85-0 |
| 9 | Magnetic stirrer | LLG LABWARE, Germany, supplier France | uni*STIRRER* 1 |
| 10 | pH meter | Hanna Instruments France, France | HI 991001 |
| 11 | Multimode patch cable | Thorlabs, USA, supplier France | M22L01 |
| 12 | Spectrometer | Ocean Optics, USA, supplier France | QE Pro ES |
| 13 | Computer | Non-applicable | |

**Table S2.** List of parameters for each molecule including starting values for the minimization algorithm.

| | | Molecule | | |
|---|---|---|---|---|
| **Coefficient** | **Starting Value** | **Fluorescein** | **FITC** | **FAM** |
| $\widetilde{K}a_1$ | $1.00 \times 10^{-2}$ | $9.25\times 10^{-4}$ | $1.90 \times 10^{-2}$ | $1.54 \times 10^{-2}$ |
| $\widetilde{K}a_2$ | $1.00 \times 10^{-5}$ | $5.66\times 10^{-6}$ | $5.26 \times 10^{-6}$ | $4.04 \times 10^{-5}$ |
| $\widetilde{K}a_3$ | $1.00 \times 10^{-6}$ | $5.46 \times 10^{-7}$ | $9.43 \times 10^{-7}$ | $2.79 \times 10^{-6}$ |
| Aa1 | 0.28 | 0.21 | 0.27 | 0.23 |
| Aa2 | 0.44 | 0.38 | 0.37 | 0.32 |
| Aa3 | 0.47 | 0.55 | 0.56 | 0.53 |
| Ac1 | 0.23 | 0.28 | 0.21 | 0.10 |
| Ac2 | 0.30 | 0.29 | 0.30 | 0.26 |
| Ac3 | 0.74 | 0.75 | 0.66 | 0.80 |
| Ad1 | 0.41 | 0.40 | 0.43 | 0.45 |
| Ad2 | 0.08 | 0.07 | 0.08 | 0.04 |
| Ad3 | 0.73 | 0.77 | 0.73 | 0.67 |
| An1 | 0.45 | 0.44 | 0.43 | 0.52 |
| An2 | 0.63 | 0.67 | 0.64 | 0.63 |
| An3 | 0.32 | 0.27 | 0.32 | 0.30 |
| Ta1 | 620 | 467.25 | 581.37 | 649.77 |
| Ta2 | 1000 | 952.70 | 985.78 | 1939.47 |
| Ta3 | 2768 | 3080.75 | 2857.80 | 3717.31 |
| Tc1 | 995 | 1111.72 | 940.00 | 1115.80 |
| Tc2 | 4091 | 4681.94 | 4699.53 | 4551.25 |
| Tc3 | 2783 | 2722.59 | 2952.21 | 3133.19 |
| Td1 | 1843 | 1835.51 | 1887.99 | 2194.87 |
| Td2 | 2978 | 2744.48 | 3032.09 | 3193.47 |
| Td3 | 1084 | 1154.68 | 1236.10 | 1149.87 |
| Tn1 | 1108 | 1129.23 | 1095.53 | 1198.29 |
| Tn2 | 4021 | 3812.93 | 3784.18 | 3724.71 |
| Tn3 | 1424 | 1231.79 | 1536.62 | 1308.06 |
| Ya1 | 507 | 507.33 | 512.23 | 513.00 |
| Ya2 | 518 | 516.72 | 523.52 | 520.97 |
| Ya3 | 532 | 523.61 | 536.07 | 533.80 |
| Yc1 | 509 | 504.95 | 527.39 | 508.53 |
| Yc2 | 570 | 570.19 | 565.38 | 573.55 |
| Yc3 | 535 | 536.12 | 553.71 | 533.70 |
| Yd1 | 534 | 536.59 | 543.35 | 533.83 |
| Yd2 | 565 | 573.52 | 578.71 | 593.04 |
| Yd3 | 511 | 511.42 | 517.58 | 512.28 |
| Yn1 | 508 | 510.47 | 512.86 | 511.12 |
| Yn2 | 544 | 544.33 | 552.35 | 549.95 |
| Yn3 | 542 | 543.47 | 544.51 | 545.73 |
